# Endotype-Guided Imaging in Chronic Rhinosinusitis: HRCT/CBCT and MRI Metrics, Structured Reporting, and Radiomics-A Systematic Review

**DOI:** 10.3390/medsci14020274

**Published:** 2026-05-28

**Authors:** Daniela Messineo, Pasquale Frisina, Elona Begvarfaj, Valeria Panebianco

**Affiliations:** 1 Department of Radiological, Oncological and Anatomical Pathology Sciences, Sapienza University of Rome, 00185 Rome, Italy; 2 Department of Sense Organs, Sapienza University of Rome, 00185 Rome, Italy

**Keywords:** high-resolution computed tomography, cone-beam computed tomography, magnetic resonance imaging, endotype, type two inflammation, osteitis, bone erosion, radiomics, structured reporting, functional endoscopic sinus surgery, orbital complications, intracranial complications

## Abstract

Chronic rhinosinusitis (CRS) is a heterogeneous inflammatory disease of the paranasal sinuses. Imaging, particularly high-resolution computed tomography (HRCT), cone-beam computed tomography (CBCT) in selected scenarios, and magnetic resonance imaging (MRI), supports diagnosis, treatment planning, and follow-up by combining anatomic mapping, disease extent, remodeling, and complication assessment. This systematic review synthesizes evidence on endotype-aware imaging in CRS and proposes a structured reporting model grounded in quantitative, management-oriented criteria. Methods: Following PRISMA 2020, the review protocol was registered in PROSPERO 2026 (CRD420261356154); we searched MEDLINE, Scopus, Embase, and Cochrane (January 2000–September 2025). Ninety-six studies were included in qualitative synthesis; 38 studies reported quantitative outcomes summarized descriptively. We evaluated HRCT/CBCT/MRI protocols, radiologic indices (Lund–Mackay, GOSS), diagnostic performance in relevant clinical domains, imaging–endotype correlations, and radiomics/artificial intelligence (AI) applications. Key Results: HRCT served as the reference modality for anatomic assessment and preoperative planning in most cohorts; CBCT was selectively used for bony evaluation with lower radiation exposure than conventional CT, albeit without reliable soft-tissue characterization. MRI was mainly applied for soft-tissue characterization, unilateral disease, and suspected complications. Radiomics/AI studies reported areas under the receiver operating characteristic curve (AUC) ranging from 0.79 to 0.93 for distinguishing type 2-high from non-type 2-high CRS, with external validation reported in a minority of studies. Conclusions: Endotype-aware imaging can support more precise, quantitative CRS management when interpreted together with clinical, endoscopic, and biomarker data. Structured reporting and radiomics may contribute to standardization, but require prospective validation and transparent reporting to enable clinical translation within multidisciplinary care pathways aligned with international guidance.

## 1. Introduction

Chronic rhinosinusitis (CRS) is a prevalent and disabling inflammatory disorder of the paranasal sinuses. Population-based estimates vary with case definition, but EPOS 2020 reports that CRS affects approximately 5–12% of the general population, with a substantial impact on healthcare utilization and productivity [1].

Beyond prevalence, CRS imposes a marked quality-of-life (QoL) burden that is frequently underestimated when disease severity is inferred only from endoscopy or sinus opacification. QoL impairment in CRS has been reported as comparable to that observed in other major chronic diseases, including congestive heart failure and chronic obstructive pulmonary disease [2].

Imaging is therefore not ancillary but pivotal in CRS, providing objective phenotyping of disease extent, mapping critical anatomic variants, and identifying complications. Contemporary appropriateness guidance positions CT and MRI as the primary imaging modalities for sinonasal disease; CT underpins preoperative planning, whereas MRI adds decisive value for soft-tissue characterization, suspected invasive fungal disease, suspected mass, and evaluation of orbital or intracranial extension [3].

Historically, sinonasal imaging evolved from plain radiography toward high-resolution CT (HRCT), enabling detailed assessment of bony anatomy, osteitis, and surgical landmarks that directly influence FESS safety and completeness. In parallel, quantitative scoring and osteitis grading were proposed to improve reproducibility and clinical interpretability; notably, the Global Osteitis Scoring Scale (GOSS) provides a structured method for capturing bony inflammatory remodeling and has been linked to CRS severity and outcomes [4].

Over the last two decades, advances in immunology have reframed CRS from a largely anatomy-centered condition to a heterogeneous disease with distinct inflammatory endotypes. Over the last two decades, advances in immunology have reframed CRS as a heterogeneous inflammatory disease characterized by multiple endotypes. Type 2-high inflammation represents the most clinically established and therapeutically actionable pattern, although mixed and non-type 2 inflammatory profiles have also been described across different populations. In particular, type 2 (T2-high) inflammation has become clinically actionable with targeted biologic therapies, strengthening the need for imaging biomarkers that can complement clinical and laboratory stratification rather than merely describe anatomy [5].

At the tissue and cellular level, type 2-high CRS is characterized by epithelial barrier dysfunction, eosinophilic inflammation, and activation of type 2 immune pathways involving IL-4, IL-5, and IL-13. These mechanisms promote polypoid remodeling and, in long-standing disease, may contribute to osteitic bony remodeling through chronic inflammatory signaling in the sinonasal microenvironment. This biological framework supports the rationale for imaging surrogates that quantify tissue burden and remodeling, while acknowledging that imaging cannot replace biomarker-based molecular endotyping [6,7].

More recently, radiomics and artificial intelligence (AI) have introduced quantitative, high-dimensional descriptors derived from routine CT/MRI, aiming to capture tissue heterogeneity and to support endotype discrimination, differential diagnosis, outcome prediction, and longitudinal monitoring. A systematic review of radiomics applied to paranasal sinus CT highlights rapid methodological expansion but also underscores the need for standardization, transparent reporting, and external validation to ensure clinical translation [8].

Two elements are particularly critical for translation: (i) radiomics feature standardization, as promoted by the Image Biomarker Standardization Initiative (IBSI), to improve reproducibility across scanners and pipelines [9] and (ii) rigorous reporting of AI studies using established checklists (e.g., CLAIM) to reduce bias and enhance interpretability and clinical trust [10,11].

Against this background, the present systematic review synthesizes evidence on HRCT, CBCT, and MRI metrics in CRS, focusing on imaging correlates of inflammatory endotypes, structured reporting, and radiomics/AI. Accordingly, our objectives were: (i) to summarize contemporary acquisition protocols and quantitative reporting metrics; (ii) to assess imaging patterns associated with biomarker-defined inflammatory endotypes and clinically relevant CRS phenotypes; and (iii) to propose a minimum structured reporting dataset to improve imaging-to-management communication. Unilateral sinonasal presentations were considered only when they informed the differential diagnosis between inflammatory CRS-related disease and neoplasia, a common real-world indication for advanced imaging.

## 2. Materials and Methods

The review was conducted and reported in accordance with the PRISMA 2020 statement [12]. The PRISMA 2020 checklist is provided in the Supplementary Materials (Table S1). Protocol and registration: The review protocol was prospectively registered in the International Prospective Register of Systematic Reviews (PROSPERO 2026; CRD420261356154). To mitigate selective reporting risk, eligibility criteria, outcomes, and synthesis decisions were prespecified before screening and are reported in detail in the Section 2 and Appendix A and Appendix B.

### Eligibility Criteria (PICO)

Population (P): Adult and pediatric patients with suspected or confirmed chronic rhinosinusitis (CRS), with or without nasal polyps (CRSwNP/CRSsNP). We also included CRS-related entities and clinically relevant differential diagnoses encountered in CRS imaging work-up (e.g., odontogenic CRS, allergic fungal rhinosinusitis, invasive fungal rhinosinusitis, and unilateral sinonasal opacification in which inflammatory disease must be differentiated from neoplasia). Studies limited to isolated rhinitis without paranasal sinus involvement were excluded.

Intervention (I): Sinonasal imaging techniques including high-resolution computed tomography (HRCT), cone-beam CT (CBCT), and magnetic resonance imaging (MRI), evaluated through standardized qualitative descriptors and quantitative scoring systems (e.g., Lund–Mackay, Global Osteitis Scoring Scale (GOSS), and olfactory cleft assessment). We included studies investigating radiomics or artificial intelligence (AI) applied to CT/MRI/CBCT data, and studies evaluating imaging-informed diagnostic or therapeutic pathways in the context of endoscopic sinus surgery (FESS) or biologic therapy monitoring when available.

Comparator (C): Nasal endoscopy, histological evaluation, clinical reference standard, inflammatory biomarkers (eosinophilia, serum IgE), and surgical or therapeutic outcomes with biological drugs.

Outcome (O): Diagnostic accuracy, imaging–endotype correlation, impact of imaging on clinical and surgical management, identification of complications, dosimetry, and inter-observer reproducibility. For AI- and radiomics-based studies, area under the ROC curve (AUC), calibration, external validation, and decision-curve analysis were also considered.

Search strategy: We searched MEDLINE (via PubMed), Embase, Scopus, and the Cochrane Library from 1 January 2000 to 30 September 2025. No language restrictions were applied during the electronic search; during full-text screening, inclusion was limited to English and Italian manuscripts due to feasibility. The complete database-specific search strings (controlled vocabulary and Boolean operators) are reported in Appendix A (Table A1) to enable replication. Reference lists of included studies and relevant reviews were hand-searched (snowballing). Clinical guidelines and society documents were consulted only to frame imaging appropriateness and were not included as primary data sources for the systematic synthesis.

**Figure 1:**
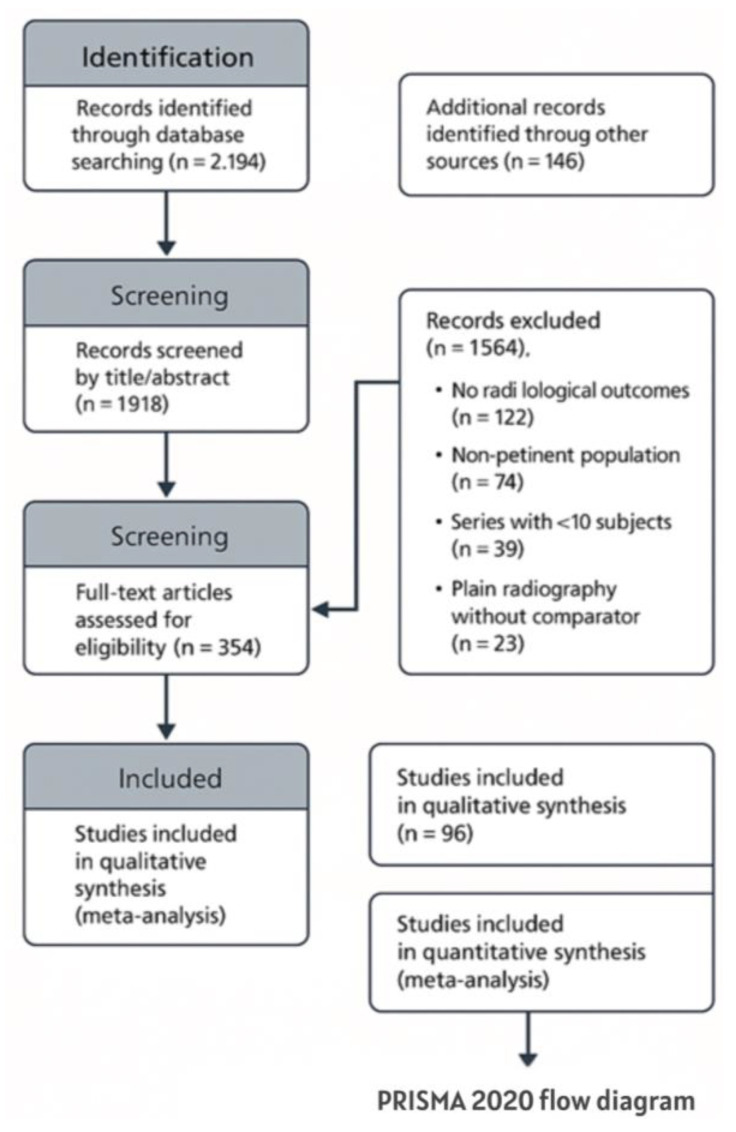
PRISMA 2020 flow diagram summarizing study identification, screening, eligibility, and inclusion.

Study selection: Two reviewers independently screened titles/abstracts and then full texts. Disagreements were resolved by discussion or by a third reviewer. The study selection process is summarized in the PRISMA 2020 flow diagram (Figure 1).

Data extraction: A pre-piloted extraction form collected study design, population characteristics (adult/pediatric), CRS phenotype and endotype definition (when reported), imaging modality and protocol parameters (including slice thickness, reconstruction kernel, MRI sequences, and dose metrics), radiologic scores (Lund–Mackay, GOSS, olfactory cleft), reference standards, outcomes, and main performance statistics.

Synthesis approach: Given heterogeneity across study designs, endpoints, and AI pipelines, we performed a structured narrative synthesis and descriptive quantitative summaries (e.g., proportions and ranges). Because of substantial methodological heterogeneity, no formal meta-analysis was performed. Quantitative findings were summarized descriptively using ranges and proportions when appropriate. Conventional imaging studies and radiomics/AI investigations were synthesized separately because of substantial differences in methodology, validation strategies, and clinical applicability. radiomics/AI performance was summarized descriptively using reported AUC values and validation approaches rather than formal quantitative pooling.

Risk of bias and study quality: Risk of bias was assessed independently by two reviewers using QUADAS-2 for diagnostic accuracy studies and ROBINS-I for non-randomized comparative studies; for radiomics/AI prediction models, reporting transparency and validation were evaluated against CLAIM/TRIPOD principles. A domain-level summary is provided in Appendix A (Table A2).

## 3. Results

### 3.1. Study Selection and Included Evidence

As summarized in the PRISMA 2020 flow diagram (Figure 1), 96 studies met the eligibility criteria and were included in the qualitative synthesis; 38 studies reported quantitative outcomes suitable for descriptive synthesis. Included evidence spanned adult and pediatric populations and a wide spectrum of CRS phenotypes; pediatric evidence was limited and should not be extrapolated to adult surgical or biologic eligibility without caution.

### 3.2. HRCT and CBCT Acquisition Protocols and Quantitative Indices

High-resolution CT (HRCT) was the reference modality for sinonasal anatomic mapping and preoperative planning in most cohorts (reported in 92% of studies). Cone-beam CT (CBCT) was used selectively for high-detail bony assessment, with reported dose reductions versus conventional CT (approximately 35–60% in the included literature), but with limited capability for soft-tissue characterization. Across studies reporting technical parameters, reconstructed slice thickness was typically submillimetric (approximately 0.8–1.0 mm), using bone algorithms and iterative reconstruction; earlier studies reported thicker reconstructions, reflecting historical technique evolution. The most frequently used staging metrics were the Lund–Mackay score and osteitis grading (commonly GOSS), primarily to standardize reporting and to support comparisons across cohorts. Anatomic variants (e.g., Onodi, Haller, Keros type) were commonly reported because of their relevance to surgical safety and planning, rather than as endotype-specific markers.

### 3.3. MRI Indications and Clinically Relevant Performance Domains

MRI was reported in 58% of studies and was mainly performed in selected scenarios, including unilateral disease, suspected tumor, suspected invasive fungal disease, and evaluation of orbital or intracranial extension. Standard T2-weighted and post-contrast T1-weighted sequences, complemented by diffusion-weighted imaging when available, supported discrimination between retained secretions/inflammatory tissue and enhancing solid components. Reported apparent diffusion coefficient (ADC) values tended to be lower in malignant lesions (mean approximately 0.85 × 10^−3^ mm^2^/s) than in inflammatory lesions (mean approximately 1.45 × 10^−3^ mm^2^/s), consistent with restricted diffusion in many malignancies.

### 3.4. Imaging Patterns Associated with CRS Phenotypes and Inflammatory Endotypes

Imaging patterns differed across CRS phenotypes and, when biomarker-defined, across inflammatory endotypes (Table A3, Appendix A). In several cohorts, type 2-high CRSwNP tended to show an ethmoid-dominant distribution, frequent olfactory cleft opacification, and more frequent imaging signs of osteitis. In contrast, CRSsNP and odontogenic CRS more often showed maxillary predominance and asymmetric mucosal thickening with dental root relationships. Distinctive patterns were also described for allergic fungal rhinosinusitis (AFRS) (central hyperattenuation on CT and low T2 signal) and for invasive fungal rhinosinusitis (IFRS) (extrasinus infiltration and the black turbinate sign on contrast-enhanced MRI). Importantly, these imaging patterns should be interpreted as supportive of a suspected endotype/etiology and integrated with clinical, endoscopic, and laboratory data; imaging alone does not define molecular endotypes. Molecular endotyping remains fundamentally biomarker-based and cannot be inferred from imaging findings alone. Imaging patterns should therefore be considered complementary phenotypic correlates that may support, but not replace, immunologic and histopathological characterization. Most included studies focused predominantly on type 2-high versus non-type 2 inflammatory patterns, whereas imaging correlates of type 1-, type 3-, or mixed inflammatory endotypes remain insufficiently characterized. Consequently, currently available imaging evidence should be interpreted as reflecting selected inflammatory profiles rather than the full immunologic heterogeneity of CRS.

### 3.5. Complications, Skull Base Defects, and Unilateral Disease

Orbital and intracranial complications were reported in a minority of surgical series (approximately 14% in the included literature), typically in clinically complicated presentations. In these contexts, HRCT was valuable for assessing bony boundaries and surgical landmarks, whereas gadolinium-enhanced MRI provided superior evaluation of orbital contents, intracranial extension, and venous structures. The highest diagnostic accuracy values reported for combined CT and MRI (up to approximately 98%) referred to selected skull base entities such as cerebrospinal fluid leaks and meningoencephaloceles, rather than to all categories of orbital/intracranial complications. Unilateral opacification and bone erosion remained key scenarios in which combined CT and MRI helped distinguish pressure remodeling and inflammatory erosion from aggressive destructive patterns suggestive of neoplasia.

### 3.6. Radiomics and Artificial Intelligence

Radiomics/AI pipelines were described in 12 studies. Most used HRCT acquisitions with submillimetric reconstructions and, less frequently, MRI with isotropic voxels. Feature extraction was commonly described as IBSI-aligned, and models typically combined first-order and texture features with machine learning classifiers (e.g., logistic regression, SVM, random forests, XGBoost). Across reported validation schemes, AUC values ranged from 0.79 to 0.93 for distinguishing type 2-high versus non-type 2-high CRS phenotypes and around 0.91 for inflammation-versus-neoplasia discrimination in unilateral disease workflows. Validation approaches varied substantially across studies and were frequently limited to internal cross-validation strategies. External validation was reported in only a minority of studies (38%), while calibration analysis and clinical utility assessment were inconsistently described. Consequently, reported performance estimates should be interpreted cautiously because of potential overfitting, methodological heterogeneity, and limited generalizability.

### 3.7. Environmental Exposure Studies (Hypothesis-Generating)

Five observational studies explored associations between air pollutants (e.g., particulate matter and nitrogen dioxide) and symptoms or inflammatory burden. These data were heterogeneous and hypothesis-generating; they did not provide sufficient evidence to define imaging schedules, but suggested a potential interaction between environmental exposure and CRS activity that warrants prospective study.

## 4. Discussion

**Figure 2:**
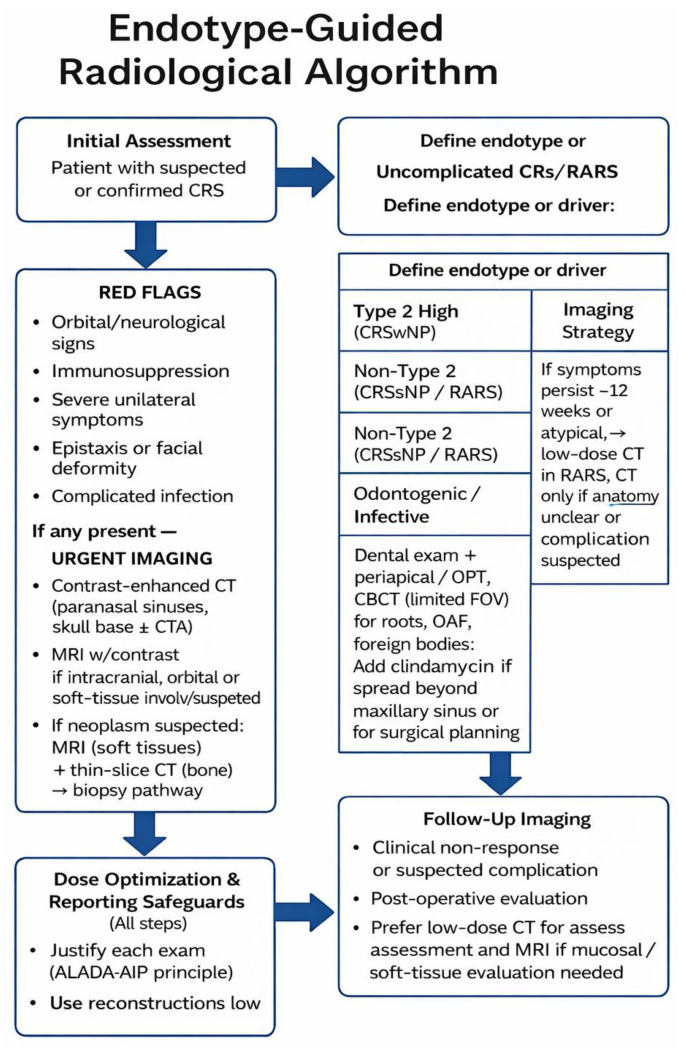
Endotype-aware imaging workflow (conceptual). Proposed decision-support framework integrating clinical criteria, nasal endoscopy, and available biomarkers to guide the appropriate use of HRCT, CBCT, and MRI. Imaging is recommended when it is expected to change management (e.g., preoperative planning, suspected complications, unilateral disease, or suspicion of invasive fungal disease or neoplasia).

Imaging is a core component of chronic rhinosinusitis (CRS) evaluation, primarily as an anatomic roadmap for endoscopic sinus surgery, an objective measure of disease extent and remodeling, and a tool to assess complications or alternative diagnoses when clinically suspected. Across the included evidence, high-resolution CT (HRCT) remains the standard technique for mapping bony anatomy, anatomic variants, and osteitic remodeling, whereas MRI adds decisive value when soft-tissue characterization is required (e.g., unilateral disease, suspected neoplasia, suspected invasive fungal disease) and when orbital or intracranial extension is a concern. Cone-beam CT (CBCT) can reduce radiation exposure in selected bony-focused questions, but it should not be considered equivalent to HRCT for soft-tissue assessment or complication work-up. Importantly, imaging findings primarily reflect disease phenotype and inflammatory burden rather than true molecular endotypes. Consequently, imaging should be interpreted together with endoscopic, histologic, and biomarker data within a multidisciplinary framework. Future research should aim to integrate imaging findings with standardized immunologic profiling, including cytokine signatures, eosinophilic biomarkers, and tissue-based inflammatory characterization. Such integration may improve understanding of how imaging phenotypes relate to the biological heterogeneity of CRS across different populations and geographic settings. A conceptual endotype-aware imaging workflow integrating clinical, endoscopic, biomarker, and imaging findings is summarized in Figure 2.

Several imaging patterns appeared to cluster with specific CRS phenotypes and inflammatory profiles. as summarized in Appendix A (Table A4). Type 2-high CRSwNP was more frequently associated with ethmoid-dominant disease, olfactory cleft involvement, and imaging signs of osteitis, whereas CRSsNP and odontogenic CRS more commonly showed maxillary-predominant and asymmetric disease. Distinctive imaging patterns for AFRS and IFRS were consistently described and remain clinically relevant because they may prompt timely multidisciplinary management (Table A4, Appendix A).

**Figure A1:**
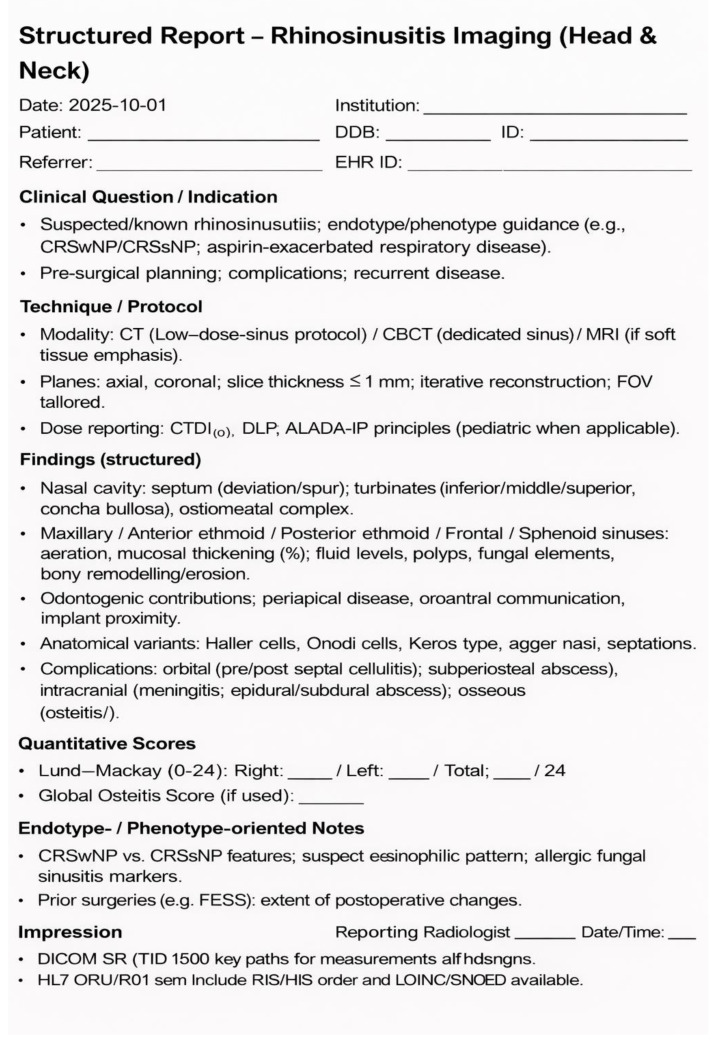
Structured radiology report template for chronic rhinosinusitis (CRS). Visual summary of the proposed standardized reporting model integrating technical parameters, critical anatomical variants, staging scores (Lund–Mackay, GOSS), olfactory cleft evaluation, and key findings for management decisions. The framework supports consistency across reports, facilitates endotype-guided interpretation, and aligns with the “findings-to-management” and IBSI-compliant reporting principles.

To translate heterogeneous imaging descriptors into reproducible, clinically actionable reports, we propose a minimum-structured reporting dataset aligned with a “findings-to-management” approach. The proposed endotype-aware reporting workflow is summarized in Figure A1 (Appendix B). For example, in a patient with ethmoid-dominant CRSwNP, olfactory cleft opacification, and imaging signs of osteitis, structured reporting may support multidisciplinary discussion regarding suspected type 2-high inflammatory burden, preoperative FESS planning, and potential eligibility for biologic therapy when integrated with biomarker and endoscopic findings. The essential elements are summarized in Table 1 and include: technical parameters and dose metrics; critical anatomic variants relevant to surgical safety; side- and sinus-specific staging (Lund–Mackay); osteitis assessment (e.g., GOSS or standardized remodeling descriptors); olfactory cleft status; and explicit red flags for invasive fungal disease, orbital/intracranial complications, or skull base defects. When radiomics/AI is applied, reports should also document the ROI definition, IBSI-compliant feature-extraction settings, calibration, and whether external validation was performed.

Complicated CRS and skull base defects remain scenarios in which the complementary strengths of CT and MRI are most clinically relevant. HRCT delineates bony boundaries and surgical landmarks, whereas gadolinium-enhanced MRI provides superior depiction of orbital and intracranial soft tissues, perineural or vascular involvement, and diffusion characteristics of suspected masses. Importantly, the highest diagnostic accuracy figures reported for combined CT and MRI (up to approximately 98%) apply to selected skull base entities (e.g., cerebrospinal fluid leak/meningoencephalocele) and should not be generalized to all orbital or intracranial complication categories.

Standardization is also needed on the surgical side to enable meaningful imaging-outcome comparisons. Beyond Lund–Mackay and osteitis grading, reporting the extent and type of endoscopic sinus surgery is increasingly recognized as a key modifier of postoperative imaging and of interpretation of recurrence. Recent classifications such as the Lamella Ostium Extent Mucosa (LOEM) system were proposed to harmonize the description of surgical extent and mucosal treatment, which may facilitate more comparable imaging and clinical endpoints across studies.

Radiomics and AI offer an additional quantitative layer that could capture tissue heterogeneity beyond human visual assessment. However, the current evidence remains heterogeneous in acquisition protocols, segmentation strategies, feature extraction settings, and validation methods. Reported AUC values for type 2-high discrimination and for inflammation-versus-neoplasia tasks are encouraging, but external validation was available in only a minority of studies, and model calibration and clinical utility were inconsistently reported. Methodological rigor (IBSI-compliant pipelines, transparent reporting in line with the CLAIM/TRIPOD principles, and external validation) is therefore essential before clinical adoption [13,14,15,16,17,18,19,20].

Several limitations should be considered when interpreting the present synthesis. First, the included studies varied widely in their definitions of CRS, endotype ascertainment (biomarkers and thresholds), imaging protocols, and outcome measures, limiting direct comparability. Second, much of the evidence was observational and single-center, with potential selection bias and incomplete reporting, particularly in radiomics/AI studies. Most evidence is derived from retrospective tertiary-center cohorts, potentially limiting generalizability to broader community-based CRS populations. Additional heterogeneity was related to differences in biomarker thresholds, CT/MRI acquisition protocols, reconstruction parameters, segmentation strategies, and radiomics pipelines, potentially affecting reproducibility and comparability across studies. Third, although no language restrictions were applied at the search stage, full-text inclusion was limited to English and Italian, potentially introducing language bias. Finally, the protocol was prospectively registered in PROSPERO; however, methodological heterogeneity across included studies remains a limitation. These issues underline the need for prospective, multicenter studies with harmonized imaging and endotyping definitions [21,22,23,24]. A small subset of observational studies explored potential associations between environmental pollutants and CRS inflammatory burden. However, available evidence remains heterogeneous and insufficient to influence imaging strategies or follow-up recommendations. This observation is hypothesis-generating and should be explored in prospective designs that can account for seasonality and treatment changes, rather than being used to define imaging indications.

**Figure 3:**
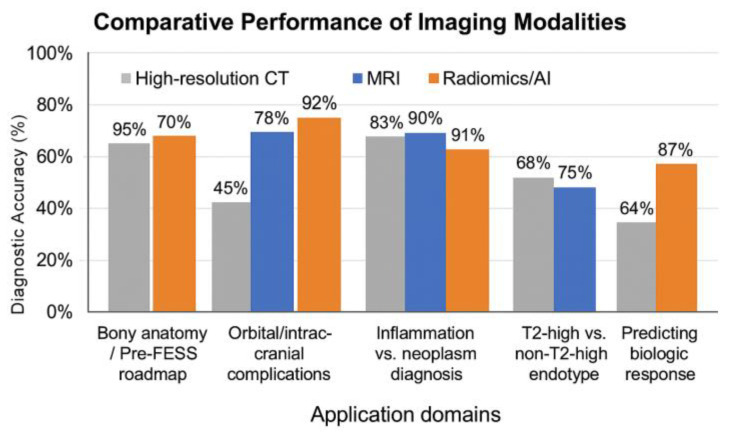
Comparative performance of imaging modalities across clinical domains in chronic rhinosinusitis (CRS).

We produced a bar chart summarizing representative accuracy ranges reported for high-resolution CT (HRCT), MRI, and radiomics/AI in selected tasks. HRCT is primarily used for bony anatomy and pre-FESS planning, whereas MRI is most informative for soft-tissue characterization, suspected complications, and inflammation-versus-neoplasia workflows. Radiomics/AI studies reported high discrimination for selected tasks (e.g., type 2-high vs. non-type 2-high phenotypes), but performance estimates vary by endpoint and validation design; therefore, values should be interpreted as task-specific rather than directly comparable across domains. Higher accuracies reported for combined CT and MRI (up to approximately 98%) refer to selected skull-base defects, such as cerebrospinal fluid leaks and meningoencephaloceles. Figure 3 summarizes representative accuracy ranges reported for high-resolution CT (HRCT), MRI, and radiomics/AI across selected clinical tasks.

## 5. Conclusions

Imaging remains essential in chronic rhinosinusitis (CRS) care for evaluating sinonasal anatomy, disease extent, inflammatory remodeling, and potential complications. High-resolution CT (HRCT) remains the first-line modality for preoperative planning and bony assessment. MRI provides complementary information for soft-tissue characterization, unilateral disease, and suspected orbital or intracranial complications. CBCT may be considered in selected bony-focused scenarios when radiation reduction is desired.

Endotype-aware interpretation of imaging may support multidisciplinary decision-making when integrated with clinical, endoscopic, and biomarker data. However, imaging findings should not be considered standalone surrogates of molecular endotypes. Radiomics and AI remain promising but currently investigational tools requiring further validation before routine clinical implementation.

Radiomics and artificial intelligence show promising potential for quantitative phenotyping and decision support. Nevertheless, broader external validation, methodological standardization, and prospective multicenter studies remain necessary before routine clinical implementation.

## Figures and Tables

**Table 1:** Proposed minimum structured reporting dataset for CRS imaging assessment.

Section	Essential Elements (Minimum Reporting Set)
Technique/dose (Essential)	CT: kVp/mAs, kernel, slice thickness ≤ 1 mm, iterative reconstruction, CTDIvol/DLP; CBCT: bone assessment only (no soft-tissue characterization); MRI: T2-weighted and T1-weighted ± gadolinium sequences; isotropic voxels when available.
Critical variants (Essential)	Onodi cell; Haller cell; Keros classification; uncinate process; lamina papyracea; cribriform plate.
Staging	Lund–Mackay score per sinus and side; ethmoid vs. maxillary-dominant distribution.
Osteitis (Optional /Advanced)	Global Osteitis Scoring Scale (GOSS) or a standardized description of cortical thickening and bony remodeling.
Olfactory cleft (Optional /Advanced)	Olfactory cleft opacification/edema; correlation with olfactory symptoms and T2-high pattern (when clinically available).
Complications (Essential)	Orbital complications (e.g., subperiosteal abscess, cavernous sinus thrombosis) and intracranial complications (e.g., empyema/abscess); flags suggestive of IFRS when present.
Radiomics (if applicable) (Optional /Advanced)	IBSI compliance statement; ROI definition (ethmoid, olfactory cleft, maxillary sinuses, bony walls); feature extraction settings (resampling/discretization); declared radiomic score (rad-score), calibration, and external validation if available.
Operational conclusion (Essential)	“Findings-to-management” statement: pre-FESS roadmap; indicate when MRI is recommended based on the clinical scenario; highlight red flags and propose follow-up imaging only when clinically indicated (i.e., when results are expected to change management).

## Data Availability

All data supporting the findings of this review are contained within the manuscript.

## Supplementary Materials

The following supporting information can be downloaded at https://www.mdpi.com/article/10.3390/medsci14020274/s1, Table S1: PRISMA 2020 Checklist.

## Abbreviations

The following abbreviations are used in this manuscript:

AFRS	Allergic Fungal Rhinosinusitis
AI	Artificial Intelligence
AUC	Area Under the Curve
CBCT	Cone-Beam Computed Tomography
CRS	Chronic Rhinosinusitis
CRSsNP	Chronic Rhinosinusitis without Nasal Polyps
CRSwNP	Chronic Rhinosinusitis with Nasal Polyps
CTA	Computed Tomography Angiography
CT	Computed Tomography
DLP	Dose Length Product
FESS	Functional Endoscopic Sinus Surgery
FLAIR	Fluid-Attenuated Inversion Recovery
FOV	Field of View
GOSS	Global Osteitis Scoring Scale
HRCT	High-Resolution Computed Tomography
IBSI	Image Biomarker Standardisation Initiative
IFRS	Invasive Fungal Rhinosinusitis
LM	Lund–Mackay Score
LOEM	Lamella Ostium Extent Mucosa
MRI	Magnetic Resonance Imaging
PM	Particulate Matter
PRISMA	Preferred Reporting Items for Systematic Reviews
ROI	Region of Interest
RARS	Recurrent Acute Rhinosinusitis
SR	Structured Reporting
T2-high	Type 2 Inflammation-High Endotype

## Appendix A

**Table A1:** Electronic search strategies (last run: 30 September 2025).

Database	Search Strategy (Verbatim)	Limits/Notes
MEDLINE (PubMed)	(“Rhinosinusitis”[Mesh] OR “Rhinosinusitis, Chronic”[Mesh] OR “chronic rhinosinusitis”[tiab] OR CRS[tiab] OR CRSwNP[tiab] OR CRSsNP[tiab] OR “nasal polyp*”[tiab]) AND (“Tomography, X-Ray Computed”[Mesh] OR CT[tiab] OR “computed tomography”[tiab] OR HRCT[tiab] OR “cone-beam computed tomography”[tiab] OR CBCT[tiab] OR “Magnetic Resonance Imaging”[Mesh] OR MRI[tiab]) AND (endotype*[tiab] OR phenotype*[tiab] OR eosinophil*[tiab] OR “type 2”[tiab] OR “type II”[tiab] OR T2[tiab] OR IL-4[tiab] OR IL-5[tiab] OR IL-13[tiab] OR IgE[tiab] OR biologic*[tiab] OR dupilumab[tiab] OR omalizumab[tiab] OR mepolizumab[tiab] OR benralizumab[tiab] OR radiomic*[tiab] OR “artificial intelligence”[tiab] OR “machine learning”[tiab] OR “deep learning”[tiab] OR “structured reporting”[tiab] OR “Lund-Mackay”[tiab] OR GOSS[tiab] OR osteitis[tiab] OR “olfactory cleft”[tiab]) AND (“1 January 2000”[Date-Publication]: “30 September 2025”[Date-Publication])	Date limits applied within query; no language filter at search stage.
Embase	(‘chronic rhinosinusitis’/exp OR ‘rhinosinusitis’/exp OR ‘chronic rhinosinusitis’:ti,ab OR rhinosinusitis:ti,ab OR CRS:ti,ab OR CRSwNP:ti,ab OR CRSsNP:ti,ab OR ‘nasal polyp’/exp OR ‘nasal polyp*’:ti,ab) AND (‘computed tomography’/exp OR ‘computed tomography’:ti,ab OR CT:ti,ab OR HRCT:ti,ab OR ‘cone beam computed tomography’/exp OR CBCT:ti,ab OR ‘magnetic resonance imaging’/exp OR MRI:ti,ab) AND (endotype*:ti,ab OR phenotype*:ti,ab OR eosinophil*:ti,ab OR ‘type 2’:ti,ab OR ‘type II’:ti,ab OR T2:ti,ab OR IL-4:ti,ab OR IL-5:ti,ab OR IL-13:ti,ab OR IgE:ti,ab OR biologic*:ti,ab OR dupilumab:ti,ab OR omalizumab:ti,ab OR mepolizumab:ti,ab OR benralizumab:ti,ab OR radiomic*:ti,ab OR ‘artificial intelligence’:ti,ab OR ‘machine learning’:ti,ab OR ‘deep learning’:ti,ab OR ‘structured reporting’:ti,ab OR ‘Lund-Mackay’:ti,ab OR GOSS:ti,ab OR osteitis:ti,ab OR ‘olfactory cleft’:ti,ab) AND [2000–2025]/py	Year filter [2000–2025]/py; no language filter at search stage.
Scopus	TITLE-ABS-KEY ((“chronic rhinosinusitis” OR rhinosinusitis OR CRS OR CRSwNP OR CRSsNP OR “nasal polyp*”) AND (HRCT OR “computed tomography” OR CT OR CBCT OR “cone beam” OR MRI OR “magnetic resonance”) AND (endotype* OR phenotype* OR eosinophil* OR “type 2” OR “type II” OR IL-4 OR IL-5 OR IL-13 OR IgE OR biologic* OR dupilumab OR omalizumab OR mepolizumab OR benralizumab OR radiomic* OR “artificial intelligence” OR “machine learning” OR “deep learning” OR “structured reporting” OR “Lund-Mackay” OR GOSS OR osteitis OR “olfactory cleft”)) AND PUBYEAR > 1999 AND PUBYEAR < 2026	Publication year 2000–2025; TITLE-ABS-KEY fields.
Cochrane Library	(chronic next rhinosinusitis OR rhinosinusitis OR CRS OR CRSwNP OR CRSsNP OR “nasal polyp*”):ti,ab,kw AND (CT OR “computed tomography” OR HRCT OR CBCT OR “cone beam” OR MRI OR “magnetic resonance”):ti,ab,kw AND (endotype* OR phenotype* OR eosinophil* OR “type 2” OR “type II” OR IL-4 OR IL-5 OR IL-13 OR IgE OR biologic* OR dupilumab OR omalizumab OR mepolizumab OR benralizumab OR radiomic* OR “artificial intelligence” OR “machine learning” OR “deep learning” OR “structured reporting” OR “Lund-Mackay” OR GOSS OR osteitis OR “olfactory cleft”):ti,ab,kw	Trials and reviews searched; year limits set in interface (2000–2025).

**Table A2:** Risk of bias and study quality appraisal (domain-level summary).

Evidence Type	Tool/Framework	Most Frequent Concerns	Interpretation Notes
Diagnostic accuracy imaging studies (CT/MRI tasks)	QUADAS-2	Patient selection (retrospective/non-consecutive sampling); heterogeneous reference standards (biomarkers/histology thresholds); incomplete blinding or unclear thresholds for index tests.	Applicability is generally acceptable for tertiary CRS settings; caution when extrapolating performance estimates to community practice.
Non-randomized comparative studies (e.g., surgical pathway or protocol comparisons)	ROBINS-I	Confounding (disease severity, prior surgery, comorbid asthma); selection bias; outcome measurement heterogeneity.	Findings should be interpreted as associative rather than causal; prospective designs are needed.
Radiomics/AI prediction or classification models	CLAIM/TRIPOD principles (plus IBSI where applicable)	Small datasets and single-center designs; variable segmentation and feature extraction settings; limited external validation; inconsistent calibration/clinical utility reporting.	Reported AUCs are task-specific and may overestimate generalizability without external validation.
Environmental exposure observational analyses	Observational quality appraisal (confounding/temporality)	Potential confounding (seasonality, treatment changes, comorbid allergy/asthma); exposure misclassification; outcomes often symptom-based rather than standardized imaging endpoints.	Hypothesis-generating; not sufficient to define imaging indications or schedules.

**Table A3:** Summary of main findings from the systematic review (2000–2025). Structured overview of imaging performance and clinically relevant correlations in chronic rhinosinusitis (CRS). Values are reported as proportions or ranges when available and are not pooled estimates.

Category	Parameter/Indicator	Modality/Group	Key Finding (Range/% Where Reported)	Notes/Caveats
Study identification	Total articles (2000–2025)	Databases + other sources	2330 records	96 included in qualitative synthesis; 38 studies reported quantitative outcomes suitable for descriptive summary.
CT HR	Utilization across studies	-	92%	Reference method for pre-FESS planning and bony anatomy assessment.
CT HR	Mean slice thickness	CT HR	Typically submillimetric	Reconstructed thickness commonly 0.6–1.0 mm; reporting varied across years and scanners.
CT HR	Lund–Mackay score	CT (HRCT/CBCT where applicable)	Frequently used	Standard 0–24 staging metric; useful for standardization but not a standalone predictor of endotype or outcomes.
CT HR	Osteitis/bony remodeling grading (e.g., GOSS)	CT (HRCT)	Reported in selected cohorts	Supports characterization of bony remodeling; definitions and thresholds varied across studies.
CT HR	Inter-reader agreement	CT HR	κ = 0.84	High reproducibility
MRI	Utilization across studies	-	58%	Mainly used for soft-tissue characterization, unilateral disease, and suspected complications.
MRI	ADC (inflammatory lesions)	MRI	1.45 ± 0.3 × 10^−3^ mm^2^/s	Free diffusion
MRI	ADC (neoplastic lesions)	MRI	0.85 ± 0.2 × 10^−3^ mm^2^/s	Marked restriction
MRI	Diagnostic performance (retained secretions vs. enhancing soft tissue)	MRI	Up to ~92% (selected studies)	Task-specific estimates; depends on sequences and reference standard.
CRS phenotypes/endotypes	Type 2-high CRSwNP (biomarker-defined when reported)	CT/MRI	Ethmoid-dominant pattern; olfactory cleft involvement	Supportive imaging pattern; endotype remains biomarker-based. Osteitis was more frequently reported in several cohorts.
CRS phenotypes/endotypes	CRSsNP/odontogenic CRS	CT (HRCT/CBCT)	Maxillary predominance; dental root relationship	Imaging supports etiologic suspicion (e.g., odontogenic source) when correlated with dental findings.
Complications	Orbital/intracranial	CT + MRI	14%	Represents proportion in selected surgical series; definitions varied.
Complications	CSF leak/meningoencephalocele	CT HR + MRI	4%	High accuracy pertains to selected skull base defects (e.g., CSF leak/meningoencephalocele).
Bone erosion	Inflammatory	CT HR/MRI	41%	Smooth margins, reactive cortical sclerosis
Bone erosion	Neoplastic	CT HR/MRI	18%	Irregular margins, cortical disruption
Radiomics/AI	Studies with a radiomics-ready pipeline	-	12% of included studies	Radiomics-ready pipelines; most used HRCT. IBSI alignment was variably reported.
Radiomics/AI	AUC (type 2-high vs. non-type 2-high discrimination)	CT/MRI	0.79–0.93	Reported across heterogeneous models; external validation in 38% of studies. Not pooled.
Radiomics/AI	AUC (inflammation vs. neoplasia discrimination in unilateral disease workflows)	CT/MRI	Around 0.91 (selected studies)	Task-specific; depends on case mix and reference standard. Not pooled.
Radiomics/AI	ΔRad-score in biologic therapy (16–24 weeks)	MRI	r = 0.71	Reported correlation in selected studies; interpretation limited by heterogeneous definitions and short follow-up.
Environmental factors	PM/NO_2_/O_3_ (seasonal burden)	-	Heterogeneous associations	Hypothesis-generating observational data; insufficient to define imaging indications or schedules.

**Table A4:** Endotype/phenotype versus imaging hallmarks (recommended reporting items).

Endotype/Phenotype	CT/MRI Hallmarks	Suggested Reporting Statement (Interpretation/Next Steps)
CRSwNP T2-high (eosinophilic)	Ethmoid-dominant pattern, olfactory cleft involvement, osteitis (elevated GOSS)	Report Lund–Mackay score per sinus/side, olfactory cleft status, and osteitis/remodeling (e.g., GOSS). Comment: ‘pattern suggestive of type 2-high inflammation; correlate with endoscopic findings and available biomarkers to support endotype assignment and treatment planning.’
CRSsNP/odontogenic	Maxillary predominance, asymmetric mucosal thickening, and dental root involvement	Indicate imaging features suggestive of an odontogenic source (e.g., periapical disease, oroantral communication) when present; recommend correlation with dental/endoscopic evaluation.
AFRS	CT: central hyperattenuation; MRI: T2-void signal	Suggest AFRS in the appropriate clinical context; describe features relevant to surgical planning and postoperative medical therapy (e.g., corticosteroids).
IFRS	Periantral or pterygopalatine infiltration; MRI black turbinate sign	If clinical suspicion of invasive fungal rhinosinusitis exists, explicitly report extrasinus spread and vascular/orbital/intracranial red flags; recommend urgent ENT evaluation. Contrast-enhanced MRI can be used to assess the extent of disease.
Cerebrospinal fluid leak/meningoencephalocele	Bony defect on HRCT; CSF signal hyperintense on T2, suppressed on FLAIR	Report the site and size of the bony defect and associated herniation/CSF signal; recommend referral to a skull base team for endoscopic repair planning.
