# Supplementary material for: Endotype-Guided Imaging in Chronic Rhinosinusitis: HRCT/CBCT and MRI Metrics, Structured Reporting, and Radiomics-A Systematic Review
Source: Med Sci (Basel). 2026 May 28;14(2):274. doi: 10.3390/medsci14020274 (PMC13303297; doi:10.3390/medsci14020274)
Supplement: Supplementary file 1 [file medsci-14-00274-s001.zip › medsci-4229830-supplementary.pdf]

Table S1: PRISMA 2020 Checklist

Item	Description	Location in manuscript
1	Identify as systematic review	Title page
2	Structured abstract	Abstract
3	Rationale	Introduction
4	Objectives	End of Introduction
5	Eligibility criteria	Section 2.1
6	Information sources	Methods
7	Search strategy	Appendix A Table A1
8	Selection process	Methods
9	Data collection	Methods
10a	Outcomes	Methods
10b	Variables	Methods
11	Risk of bias	Methods + Table A2
12	Effect measures	Methods
13a	Synthesis eligibility	Methods
13b	Data preparation	Methods
13c	Presentation	Tables A1–A3
13d	Synthesis	Results 3.2–3.7
13e	Heterogeneity	Discussion
13f	Sensitivity analysis	Not applicable
14	Reporting bias	Discussion
15	Certainty	Not performed
16a	Study selection	Figure 1
16b	Excluded studies	Figure 1
17	Study characteristics	Tables A3-A4
18	Risk of bias	Table A2
19	Results	Results
20a	Summary	Results 3.1
20b	Statistical synthesis	Descriptive only
20c	Heterogeneity	Discussion
20d	Sensitivity	Not applicable
21	Reporting bias	Discussion
22	Certainty	Not performed
23a	Interpretation	Discussion
23b	Limitations evidence	Discussion
23c	Limitations review	Discussion
23d	Implications	Discussion + Conclusions
24a	Registration	PROSPERO CRD420261356154
24b	Protocol	PROSPERO
24c	Amendments	Not reported
25	Support	Funding
26	Competing interests	Conflicts of Interest
27	Data availability	Data Availability Statement
